# The role of mutations at codons 32, 47, 54, and 90 in HIV-1 protease flap dynamics

**DOI:** 10.15190/d.2014.19

**Published:** 2014-12-31

**Authors:** Poorvi Chordia, Tamaria G. Dewdney, Bradley Keusch, Benjamin D. Kuiper, Kyla Ross, Iulia A. Kovari, Rodger MacArthur, Hossein Salimnia, Ladislau C. Kovari

**Affiliations:** Department of Biochemistry and Molecular Biology, Wayne State University School of Medicine, Detroit, Michigan, USA; Department of Infectious Diseases, Wayne State University School of Medicine, Detroit, Michigan, USA; Department of Pathology, Wayne State University School of Medicine, Detroit, Michigan, USA

**Keywords:** HIV-1 protease, molecular dynamics, drug resistance, protease mutations

## Abstract

Treatment of Human Immunodeficiency Virus remains challenging due to the emergence of drug resistant strains under the selective pressure produced by standard anti-retroviral therapy. To explore the structural mechanisms of drug resistance, we performed 40 ns molecular dynamics simulations on three multi-drug resistant HIV-1 protease clinical isolates from patients attending an infectious diseases clinic in Detroit, MI. We identify a novel structural role for the I47V, V32I, I54M and L90M major resistance mutations in flap opening and closure of MDR-PR isolates. Our studies suggest I47V is involved in flap opening and the interaction between I47V and V32I tethers the flaps to the active site. Also, I54M and L90M may be responsible for asymmetric movement of the protease flaps. These findings can be utilized to improve drug design strategies against MDR HIV-1 PR variants.

## INTRODUCTION

The high viral replication rate and error-prone reverse transcriptase leads to the emergence of drug resistant human immunodeficiency virus variants under the selective pressure of anti-retroviral therapy^1,2^. The current standard treatment of HIV utilizes HAART where the PIs represent a key class of drugs^3^. PIs have a higher barrier of resistance relative to other classes of inhibitors, as multiple protease mutations are needed for a patient to develop resistance^4-6^. Although there are nine United States FDA approved PIs, some first generation inhibitors (e.g. indinavir, saquinavir, and nelfinavir) are considered obsolete by clinicians due to emergence of drug resistance or from their side effects^3^. Second generation PIs, especially darunavir and tipranavir, remain potent against mutant viral strains that are resistant to first generation PIs^7,8^. With the availability of second generation PIs, treatment of patients with MDR HIV is possible and can result in full viral suppression^9^. Many factors^10-25^, including mutations in the viral polyprotein (particularly in Gag and Env)^21^, the development of cross-resistance^10^, and the high mutability of the HIV-PR^11^ continue to produce viral strains that are resistant to second generation PIs^12,13^. Therefore, novel and potent new drugs are required for the treatment of multi-drug resistant HIV infection.

This study utilized molecular dynamics simulation techniques in order to better understand the molecular mechanisms by which MDR-PRs are able to evade inhibition by potent PIs. Clinical MDR-PR isolates were obtained from three patients at the Wayne State University Infectious Disease Clinic in Detroit, MI. On a boosted PI regimen, these patients had a low CD4 cell count and high HIV viral load, indicating treatment failure. Genotypic and virtual phenotypic analysis of the protease genes showed multiple major and accessory drug resistance mutations which conferred extensive resistance to the nine FDA approved PIs.

**Figure 1:**
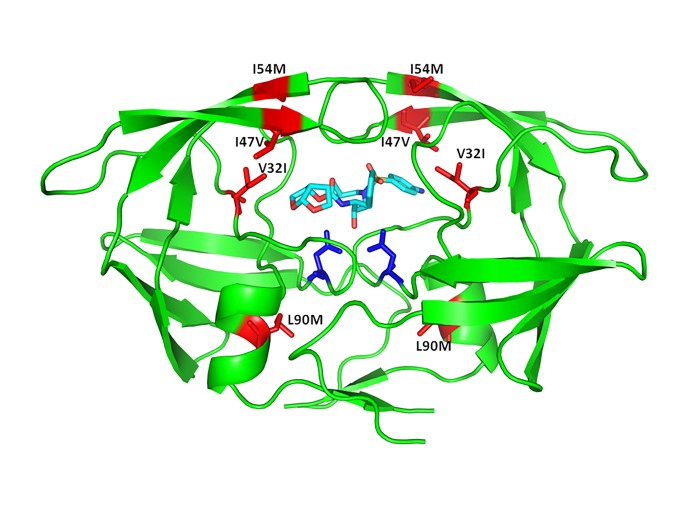
Structure of the HIV-1 PR in complex with the PI darunavir The HIV-1 PR is shown as a cartoon in green. The key residue mutations contributing to flap dynamics are shown in red. The catalytic aspartic acid residues are shown in blue. Darunavir is bound to the active site in cyan.

The HIV PR is a homodimer containing 99 amino acid residues per monomer^14^. The DetMDRs differ from isolates previously studied by our group in that these contain the major drug resistance mutations L33F, I47V, I50V, I54M, L76V, V82I/F, and I84F not present in the previous cohort^7,16-18^ (**Table 1**). The DetMDRs also contain previously identified non-polymorphic accessory mutations L10V/G, V11I, I13V, K20T/R, L33F/I/M, K43T, F53L, A71L, T74P, and L89V. To understand the molecular mechanisms of resistance of the MDR HIV PR, 40 ns molecular dynamics simulations of apo and complexed DetMDRs were performed. These studies provide insight into the structural changes that lead to multidrug resistance; in particular, we identify previously unreported roles for the I47V, V32I, I54M, L90M mutations in protease flap dynamics (**Figure 1**).

**Table 1:** Sequence alignment of WT, MDR-769, DetMDR1-3

NL4-3	PQITLWKRPL VTIKIGGQLK EALLDTGADD TVLEEMNLPG RWKPKMIGGI GGFIKVRQYD QILIEICGHK AIGTVLVGPT PVNIIGRNLL TQIGCTLNF
MDR-769	PQITLWKRPI VTIKIGGQLK EALLDTGADD TVLEEVNLPG RWKPKLIGGI GGFVKVRQYD QVPIEICGHK VIGTVLVGPT PTNVIGRNLM TQIGCTLNF
DetMDR1	PQITLWKRPV ITVKIAGQLK EALLDTGADD TIFEEMNLPG RWTPKIVGGI GGFMKVRQYD QIPIEICGHK LVGPVLVGPT PTNVIGRNMM TQLGCTLNF
DetMDR2	PQITLWKRPV VTVKVGGQLF EALLDTGADD TVFEEINLPG RWKPKIVGGI GGFVKVRQYD QILIEICGKK IISTVLVGPT PVNVIGRNTL TQMGCTLNF
DetMDR3	PQITLWKRPF VTVKIGGQLI EALLDTGADD TIFEEMNLPG RWKPKIIGGI GGFLKVRQYD QILIEICGHK AIGTVVVGPT PVNVIGRNML TQIGCTLNF

## MATERIALS AND METHODS

### MDR genotype and virtual phenotype of Detroit isolates

Sequence comparisons of the Detroit isolates highlight the differences as well as the common mutations present in each isolate (**Table 1**). All three isolates contain the L33F, M46I, and I84V major drug resistance mutations and the I13V accessory mutation. The protease sequences of the Detroit isolates were submitted for virtual phenotype predictions with vircoTYPE (**Table 2**). This software uses an input sequence, along with known information regarding resistance mutations to calculate quantitative levels of phenotypic susceptibility to the entire class of PIs19. Clinicians use this information as a guide when prescribing individualized therapy for their HIV infected patients. The vircoTYPE predictions of the Detroit isolates suggest that DetMDR1 is highly resistant to all nine FDA approved PIs. DetMDR2 is susceptible to DRV with reduced susceptibility to IND, SAQ, LPV, and ATV. DetMDR3 is predicted to be susceptible to SAQ and TPV with reduced susceptibility to LPV, ATV, and IND.

**Table 2:** Virtual phenotype predictions of the Detroit Isolates with VircoType suggest multidrug resistant phenotype ****minR****: Minimal Response; redR: Reduced Response; ****maxR****: Maximal Response; **X/r** = ritonavir boosted

Isolate	IDV	IDV/r	NFV	SQV/r	FPV/r	LPV/r	ATV/r	TPV/r	DRV/r
**Isolate 1**	87.3 (minR)	87.3 (minR.)	65.2 (minR)	49.0 (minR)	68.5 (minR)	113.2 (minR)	79.2 (minR)	123.2 (minR)	208.2 (minR)
**Isolate 2**	22.5 (redR)	22.5 (redR)	34.1 (minR)	17.1(redR)	31.2 (minR)	27.5 (redR)	28.9(redR)	16.1(minR)	9 (maxR)
**Isolate 3**	8.8 (minR)	8.8(redR)	10.1(minR)	0.9(maxR)	70.0(minR)	39.4 (redR)	17.8(redR)	0.7(maxR)	151.6 (minR)

### Simulation preparation and molecular dynamics

The crystal structure of the protease-darunavir complex (PDB: 3D20)24 was used to build a structure based model that include the DetMDR mutations and the NL4-3 wild type template (**Table 1**). All residues were mutated using the mutagenesis tool in PyMol20. Based on the catalytic mechanism of HIV-1 protease, Asp 25 was assigned a protonated state while Asp 25’ was assigned a deprotonated state to prevent ionic repulsion during the simulation. All histidine residues were assigned a neutral charge. Crystallographic water molecules were retained in the initial setup and the ligand was removed for the apo simulations. The coordinates for the ligands DRV, ATV, and LPV were obtained from structures in the PDB (3D20, 3OXX, and 2O4S)^24,25^.

The MD simulations were performed using the parallel computing program Scaling NAno Molecular Dynamics (NAMD) V. 2.9^23^. The PR complex models were solvated in a 12Å water box using TIP3P models for water molecules^22^. The cutoff for non-bonded interactions was 10 Å. The Particle Mesh Ewald method was implemented to calculate long-range electrostatic interactions. The systems were energy minimized using 20,000 steps of the conjugate gradient method and gradually heated from 0K to 300K. Simulations were conducted in the isobaric-isothermal ensemble at 300K and 1.0 atm (NPT ensemble) for 40 ns using the CHARMM all-atom force field 36 and a time step of 2 fs. Langevin dynamics ensured a constant temperature, with a damping coefficient of 5 ps-1. The Nose-Hoover piston method maintained a constant pressure of 1 atm.

### Data analysis of molecular dynamics simulation

Trajectories of MD simulation were visualized and analyzed using the Visual Molecular Dynamics (VMD) program v 1.91. The RMSD values were calculated using the VMD RMSD trajectory plug-in. The hydrogen bonding network was calculated using the H-bond tool in VMD, with 3.0 Å distance and 20 degree angle cutoffs. Structure figures were made using PyMol.

## RESULTS

### Mutations in the Detroit isolates reveal alternative protein dynamics

**Figure 2:**
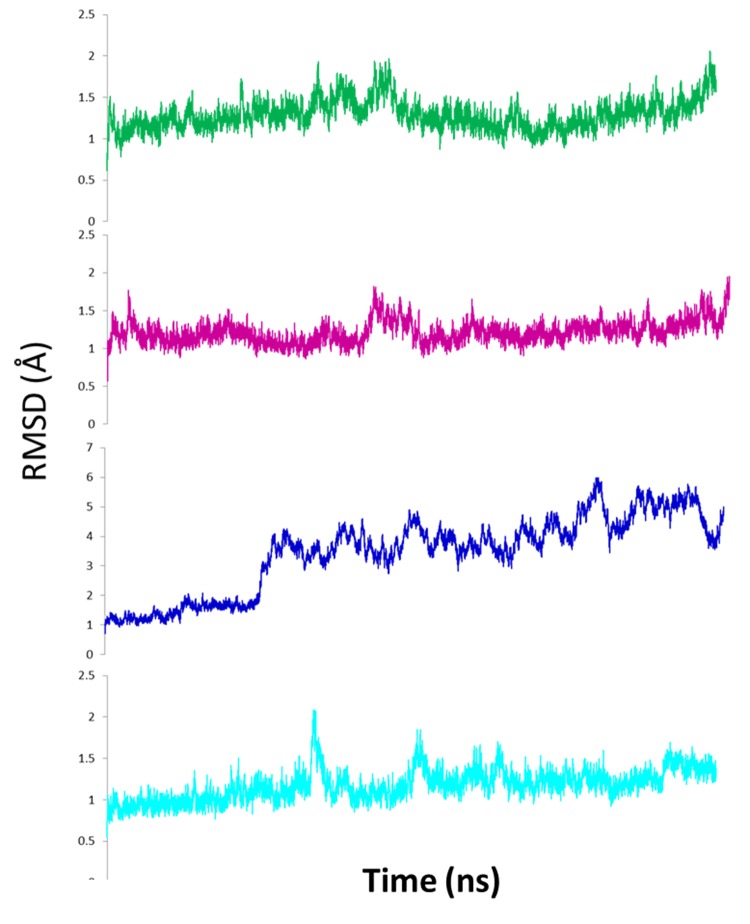
Root mean square deviation (RMSD) of Cα positions of the apo HIV-1 protease isolates Wild-type is shown in green, DetMDR1 in pink, DetMDR2 in blue and DetMDR3 in cyan. ****Note:**** DetMDR2 plot (blue) has altered y-axis scale.

RMSD analysis of the apo structure of the DetMDR isolates show increased fluctuation of all three isolates compared to the WT PR (****Figure 2****). DetMDR1 shows only a brief increase in flexibility while DetMDR2 shows a sharp increase followed by sustained elevation in RMSD for the rest of the trajectory. DetMDR3 displays short bursts of increased flexibility but no sustained changes in RMSD. These data suggest a large conformational change in DetMDR1 and DetMDR2 but not in DetMDR3 or the WT.**

### V32I interacts with I47V to tether the protease flaps in a closed conformation

**Figure 3:**
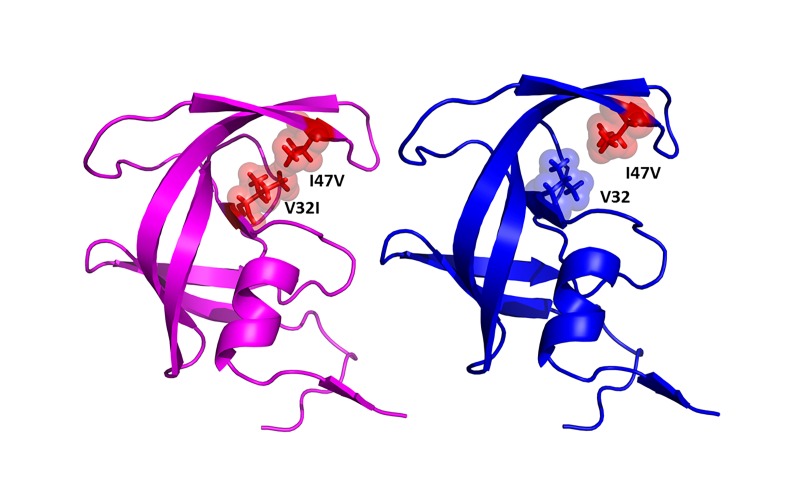
Change in the van der Waals volume induced by the drug resistance mutations I47V and V32I The left panel shows DetMDR1; the right panel shows DetMDR2.

Visual analysis of the trajectory shows that these increases in RMSD are due to opening of the flaps in DetMDR1 and DetMDR2. In DetMDR1 the flaps open and then close. In contrast, the flaps of DetMDR2 open and stay open for the remainder of the trajectory. The WT and DetMDR3 flaps do not open during these simulations. These differences in flap movement correspond to the presence or absence of particular mutations. I47V likely contributes to flap opening as it is present in both isolates 1 and 2 where the flaps open but not in DetMDR3 where the flaps do not open. However, vdW interactions between the V32I and I47V mutations cause flap closure in HIV-1 PR (**Figure 3**). These vdW interactions could explain the flap opening followed by quick flap closure observed in DetMDR1, while the absence of V32I in DetMDR2 removes this interaction and cause the flaps to remain open. Similarly, the WT and DetMDR3 have V32 and I47 at these loci and therefore the vdW interactions in the WT and DetMDR3 are maintained casing the flaps to remain closed.**

### I54M in combination with L90M plays a role in asymmetric flap movement

**Figure 4:**
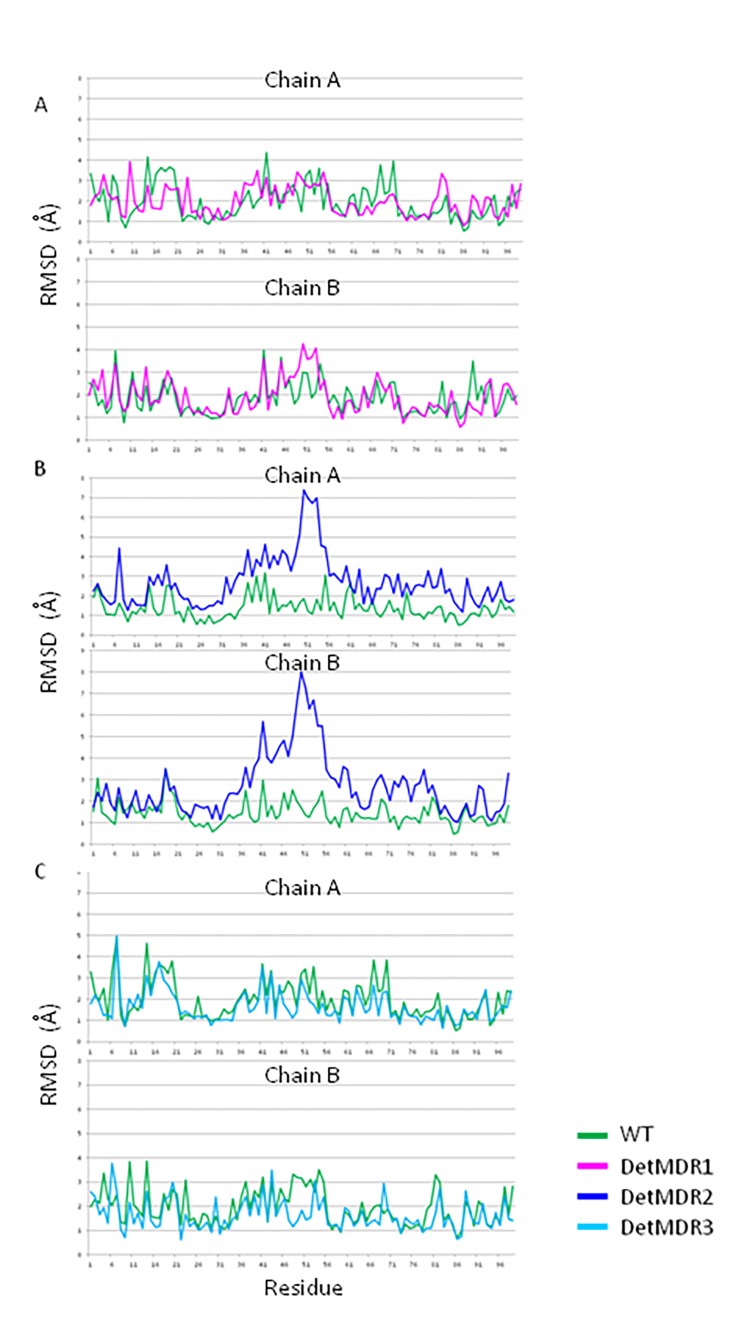
RMSD per residue of the uncomplexed HIV-1 protease isolates compared to WT reveal alternate flap dynamics of WT and Detroit MDRs ****A.**** DetMDR1 ****B.**** DetMDR2 and ****C.**** DetMDR3. The flap regions corresponding to residues 47-54 show increase in RMSD on both protease chains in DetMDR2 and only on chain B of DetMDR1

I54M and L90M are associated with asymmetric movement in DetMDR1 corresponding to opening of the flaps. The RMSD of L90M on chain B is different than that of chain A. In DetMDR1 the asymmetric movement of the flaps as indicated by the differences in RMSD at each residue show a difference in residues 47-54 on chain B compared to chain A (****Figure 4****). Movement of the residues in this region was symmetric in DetMDR2 where the residue fluctuation per residue is conserved between both chains of the PR.**

### Darunavir, atazanavir and lopinavir binding stabilize the HIV-1 protease flaps

**Figure 5:**
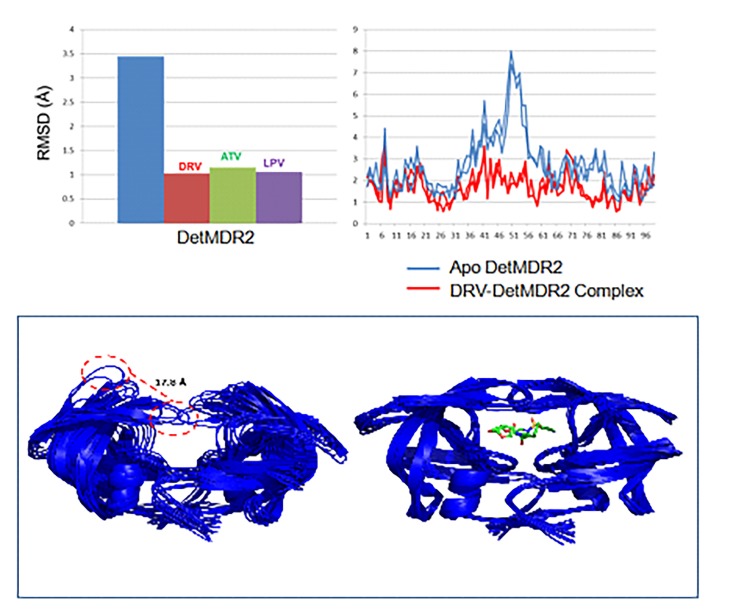
Darunavir, atazanavir and lopinavir binding stabilize the HIV-1 protease flaps The top left panel shows the average RMSD of DetMDR2 either alone (blue) or in complex with DRV (red) ATV (green) and LPV (purple). The RMSD per residue is shown in the top right panel. The bottom left panel is representative of the 40ns MD simulation using frames post energy minimization and at 4ns intervals of uncomplexed and DRV complexed DetMDR2, respectively aligned by the Cα positions. The red dashed lines indicate a 17.8Å movement of the Cα of Ile50 of the protease flap. This figure was made in PyMol.

The opening of the flaps observed in the apo simulations of DetMDR1 and DetMDR2 did not occur in the drug complex simulations. Drug binding maintains a closed flap conformation in DetMDR2. This suggests that the structural mechanism of drug resistance in these isolates is not due to changes in protein flexibility (**Figure 5**).**

### Mutation-induced changes to the hydrogen bonding network alters inhibitor conformation

In addition to the altered protein dynamics, there is a change in the hydrogen bonding interactions in the WT compared to the MDR complex (*data not shown*). DetMDR1 has 2.3%, 0.8% and 19.2% reduced hydrogen bond formation (compared to the WT) when complexed with DRV, ATV, and LPV respectively. Interestingly, DetMDR2 and DetMDR3 have increased hydrogen bond formation when in complex with DRV and ATV and a 21% and 36% decrease in hydrogen bond formation when in complex with LPV. The increase in hydrogen bonding is likely due to an alteration in the residues involved in an interaction with the PI. While changing the binding pocket may increase hydrogen bond formation, this also alters the conformation of the inhibitor.

## CONCLUSION

Three multi-drug resistant HIV-1 protease clinical isolates were selected from patients attending the Wayne State University infectious diseases clinic and failing antiviral treatment therapy on PI based regimens. To explore the structural mechanisms resulting in treatment failure, we performed 40ns MD simulations on the Detroit MDR series. Our results indicate a novel structural role for the I47V, V32I, I54M and L90M resistance mutations.

The V32I and I47V mutations play a structural role in tethering the flaps to the active site. Sequence analysis comparisons of the DetMDR protease isolates showed that DetMDR2 does not contain the V32I and I47V mutation combination. It contains only the I47V mutation and without the V32I mutation there is a loss in vdW contact volume between these two residues. Analysis of this apo PR trajectory displayed a marked increase in RMSD when compared to the WT, and a visual inspection of the trajectory as well as per-residue RMSD analysis confirmed that this is due to the flaps opening. Therefore, we postulate that I47V is responsible for flap opening. However, V32I is a compensatory mutation that may be responsible for tethering the flaps to the active site through its contacts with I47V. Therefore, if I47V is the only mutation it may be responsible for promoting flap opening through the disruption of the vdW interactions between residues 32 and 47.

I54M and L90M may be responsible for asymmetric movement of the protease flaps. This mutation combination is only present in DetMDR1 which is the only protease in the series in where the flaps asymmetrically open and then close after 4 ns. The mechanism explaining asymmetric flap opening will be explored further in future characterization studies of the HIV-1 protease isolates.

The role of these structural changes in drug resistance was investigated with the Detroit isolates in complex with the protease inhibitors ATV, DRV, and LPV. These results suggest that drug resistance occurs through alternate hydrogen bonding interactions in each of the mutants. Both the alternate hydrogen bonding networks and the flexibility of the flaps are stabilized with the protease inhibitors bound to the active site.

In summary, this work addresses the role of mutations at codons 32, 47, 54, and 90 in HIV-1 protease. We have demonstrated the role of these mutations in the altered flap dynamics. Finally, the reported findings can be utilized to enhance drug design strategies against MDR HIV-1 PR variants.

- **I47V HIV-1 mutation is involved in flap opening and the interaction between I47V and V32I tethers the flaps to the active site**.
- **These findings are important in drug design strategies**.
